# Chronic nerve health following implantation of femoral nerve cuff electrodes

**DOI:** 10.1186/s12984-020-00720-3

**Published:** 2020-07-14

**Authors:** Max J. Freeberg, Gilles C. J. Pinault, Dustin J. Tyler, Ronald J. Triolo, Rahila Ansari

**Affiliations:** 1 Department of Biomedical Engineering, Case Western Reserve University, Cleveland, OH USA; 2 Advanced Platform Technology (APT) Center, Cleveland, OH USA; 3 Department of Surgery, Case Western Reserve University, Cleveland, OH USA; 4 Louis Stokes Cleveland VA Medical Center, Cleveland, OH USA; 5 Department of Neurology, Case Western Reserve University, Cleveland, OH USA

**Keywords:** Chronic nerve health, Electromyography, Rehabilitation, peripheral nerve cuff electrodes, Electrodiagnostics and neuromuscular diseases, Electrical stimulation, Spinal cord injury

## Abstract

**Background:**

Peripheral nerve stimulation with implanted nerve cuff electrodes can restore standing, stepping and other functions to individuals with spinal cord injury (SCI). We performed the first study to evaluate the clinical electrodiagnostic changes due to electrode implantation acutely, chronic presence on the nerve peri- and post-operatively, and long-term delivery of electrical stimulation.

**Methods:**

A man with bilateral lower extremity paralysis secondary to cervical SCI sustained 5 years prior to enrollment received an implanted standing neuroprosthesis including composite flat interface nerve electrodes (C-FINEs) electrodes implanted around the proximal femoral nerves near the inguinal ligaments. Electromyography quantified neurophysiology preoperatively, intraoperatively, and through 1 year postoperatively. Stimulation charge thresholds, evoked knee extension moments, and weight distribution during standing quantified neuroprosthesis function over the same interval.

**Results:**

Femoral compound motor unit action potentials increased 31% in amplitude and 34% in area while evoked knee extension moments increased significantly (*p* < 0.01) by 79% over 1 year of rehabilitation with standing and quadriceps exercises. Charge thresholds were low and stable, averaging 19.7 nC ± 6.2 (SEM). Changes in saphenous nerve action potentials and needle electromyography suggested minor nerve irritation perioperatively.

**Conclusions:**

This is the first human trial reporting acute and chronic neurophysiologic changes due to application of and stimulation through nerve cuff electrodes. Electrodiagnostics indicated preserved nerve health with strengthened responses following stimulated exercise. Temporary electrodiagnostic changes suggest minor nerve irritation only intra- and peri-operatively, not continuing chronically nor impacting function. These outcomes follow implantation of a neuroprosthesis enabling standing and demonstrate the ability to safely implant electrodes on the proximal femoral nerve close to the inguinal ligament. We demonstrate the electrodiagnostic findings that can be expected from implanting nerve cuff electrodes and their time-course for resolution, potentially applicable to prostheses modulating other peripheral nerves and functions.

**Trial registration:**

ClinicalTrials.govNCT01923662, retrospectively registered August 15, 2013.

## Background

Electrical stimulation of peripheral nerves can restore or modulate functions of the somatic and autonomic peripheral nervous system. Somatic interfaces can modulate several functions ranging from prostheses restoring movement following central nervous system injury [1–3] to those restoring sensation and controlling prostheses following extremity amputation [4–7]. Surgically implanted neuroprostheses can restore function to individuals with spinal cord injury (SCI) by eliciting contractions of the otherwise paralyzed muscles via electrical stimulation of peripheral motor nerves [1, 8–12]. These systems can restore numerous functions—including walking, cycling, and bladder function—by interfacing with the nervous system at the spinal cord [13–17], at the anterior roots [18–22], at peripheral nerves [1, 3], and at the points of innervation in muscles [3, 12, 23], or some combination thereof.

Nerve-based electrodes have advantages over other electrode designs [24, 25], including robust, stable recruitment, and improved functional outcomes [1, 26]. These electrodes may be penetrating or non-penetrating. While penetrating electrodes offer the potential of higher selectivity [27–29], non-penetrating nerve cuff electrodes (NCEs) on nerves in the upper [2, 4, 5, 30] and lower [3, 26, 31–35] extremities have been operational and stable for more than 11 years post-implantation [31] in terms of stimulation threshold and functional output over time [2, 26]. To date, the effects of both implantation and chronic use of NCEs on neurophysiology and muscle innervation have not been examined with established clinical measures, including both nerve conduction studies (NCS) and needle electromyography (EMG). This dearth of neurophysiological outcomes and lack of baseline electrodiagnostic changes in well-functioning neuroprostheses make it difficult to gauge the impact of novel NCE designs, implant locations, surgical approaches, and rehabilitation paradigms on nerve health.

**Fig. 1:**
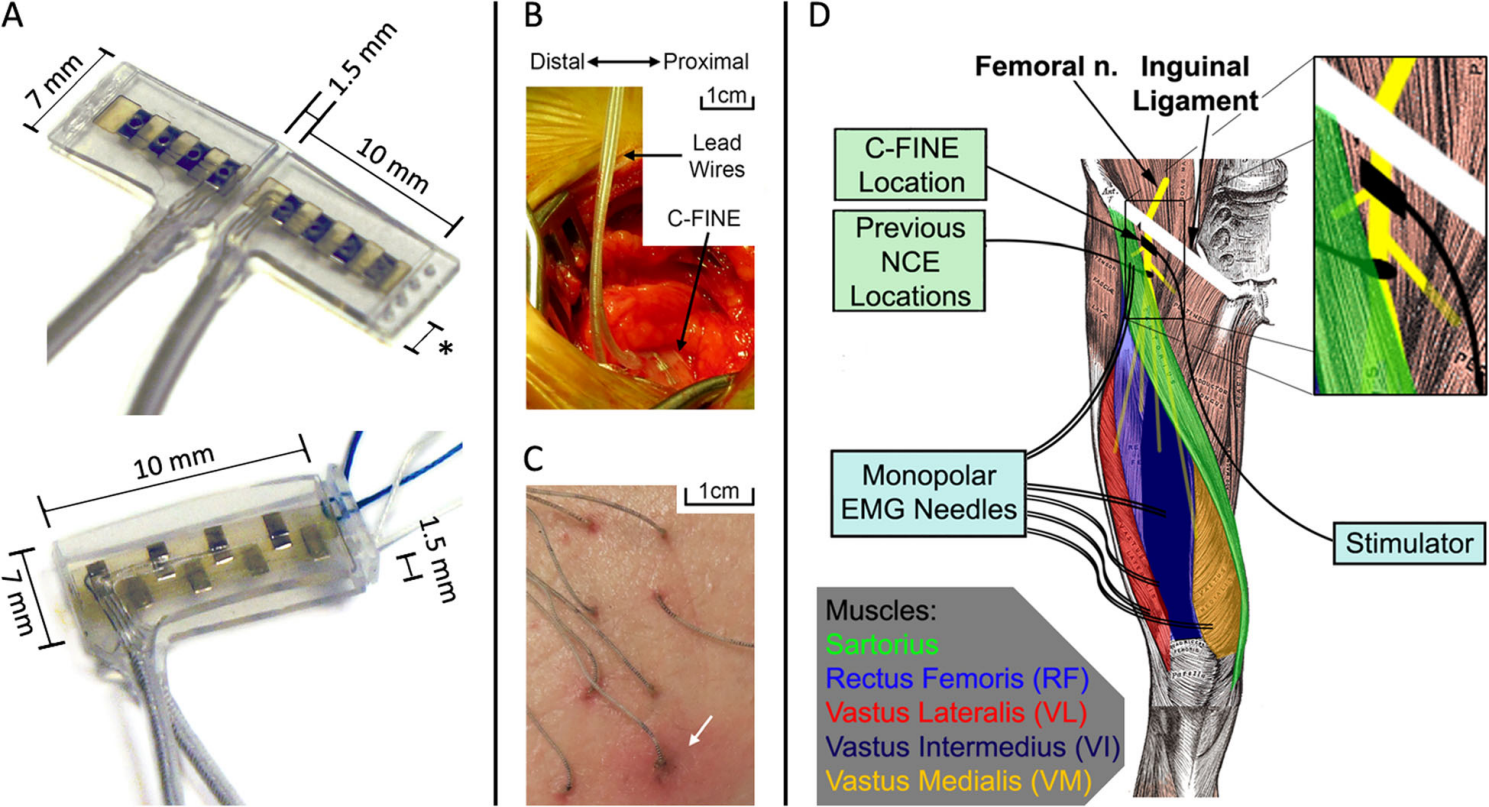
C-FINEs, implantation on femoral nerves, percutaneous leads, and intraoperative EMG. **a** Example 8-contact C-FINE in open (top) and closed (bottom) configurations. Asterisk indicates flexible edges of C-FINE. **b** Images of C-FINE placed around femoral nerve (top). **c** Percutaneous leads approximately 3 months after implantation (bottom). White arrow indicates an area of mild, occasional erythema at one of the indwelling leads. **d** Intraoperative EMG collection (light blue boxes) and implant location compared to previous implants [1] (light green boxes and inset at top right). Note proximity of NCE to inguinal ligament to be implanted proximal to the first branches off the femoral nerve

As NCE designs include higher contact densities [36] they are able to achieve sufficient selectivity to be implanted on proximal nerve trunks [37]. The flat interface nerve electrode gently reshapes peripheral nerves to increase accessible surface area for multiple contacts, allowing for better access to distinct populations of nerve fascicles [36]. The 8-contact FINE is sufficiently selective to separate hip flexors from knee extensors [37] when implanted proximal to the first branch off the femoral nerve, approximately 1–2 cm distal to the inguinal ligament [38]. The inguinal ligament is a possible site of femoral nerve entrapments, especially during hip flexion and rotation [39–42]. The composite flat interface electrode (C-FINE, Fig. 1a) [43] was developed to be a small and flexible reshaping NCE to accommodate the stresses and strains in such locations. Yet, the acute and chronic consequences of the C-FINE on neurophysiology, especially in novel implant locations (e.g., close to the inguinal ligament) have not been established.

Numerous elements of a neuroprosthesis, such as NCE design, lead routing, implant location, and surgical approaches, may affect nerve health. Neurophysiological and electrodiagnostic changes can also be caused by intraoperative nerve manipulation, perioperative positioning [41] and edema [44], and nerve traction. These changes can range from temporary demyelination and neurapraxia to varying degrees of axonotmesis [45], with a wide range of prognoses [46]. The neurophysiologic consequences of neuroprosthesis design changes have been extrapolated from benchtop and animal models [43, 47, 48] and then tracked chronically through observations of neuroprosthesis performance [1, 30]. With myriad potential etiologies and sequelae, it is crucial to grade neurophysiological changes following NCE implantation using well-established electrodiagnostic studies in chronic implant recipients. This is especially important in subjects with SCI who have limited clinical exams at baseline.

**Fig. 2:**
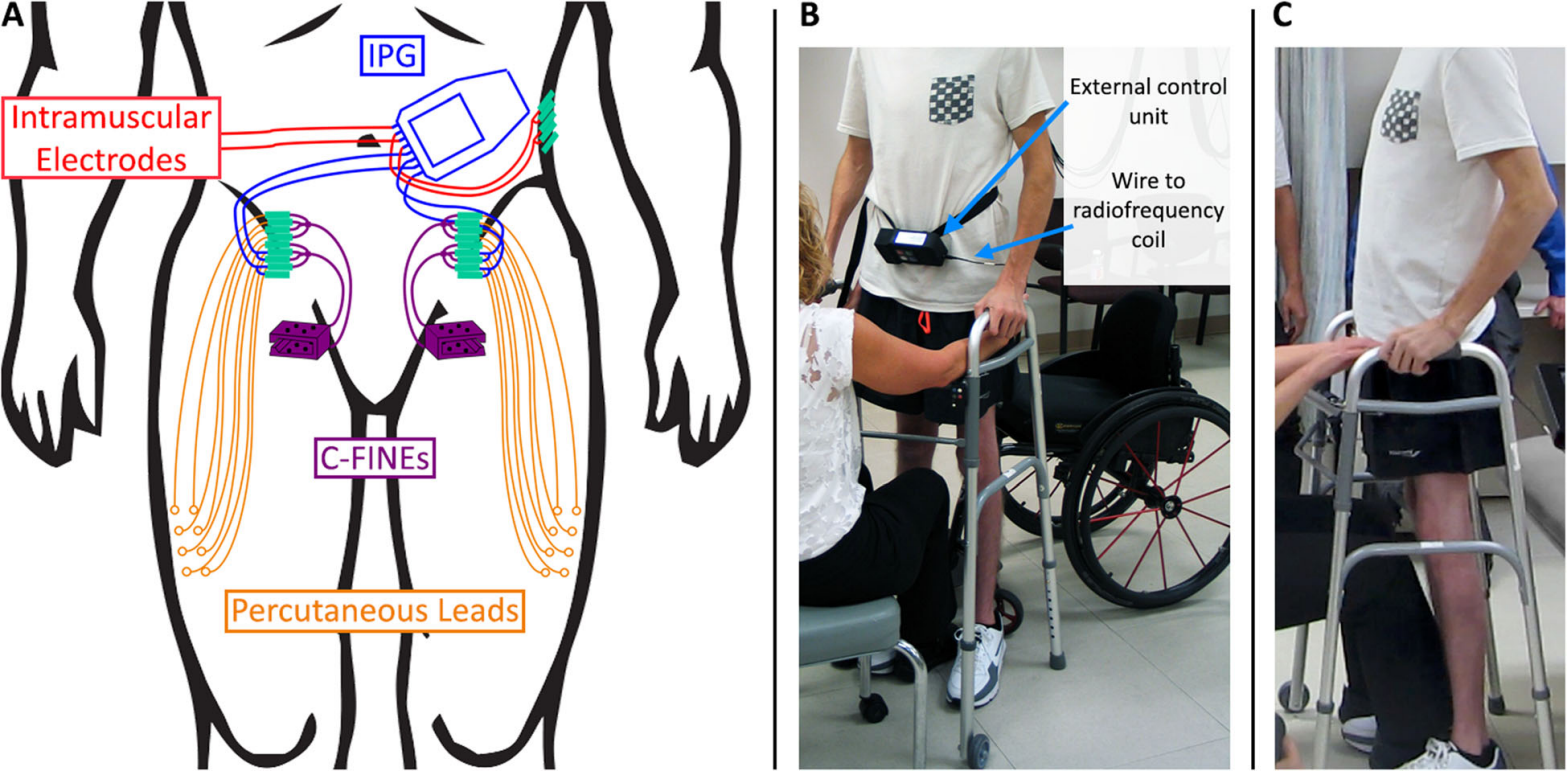
Implant diagram and C-FINE recipient standing using implanted neuroprosthesis. **a** Lead routing showing C-FINEs (purple) plugged into connectors (green) which are either connected to percutaneous leads (percutaneous phase, orange wires) or an IPG (standing phase, blue wires). Red wires indicate IPG connections to intramuscular electrodes which are used to stimulate muscles not innervated by the femoral nerves and are required for standing. **b** and **c** Pictures show subject standing from his wheelchair taken from his front (**b**) and from his side (**c**). External control unit communicates with the IPG via a pair of radiofrequency coils. A physical therapist is spotting the C-FINE recipient but is not providing support, as later confirmed by force plate measurements

We performed the first human trial evaluating the short and long-term health of nerves implanted and stimulated with NCEs through electrodiagnostic studies preoperatively, intraoperatively, and postoperatively over 1 year. We tracked femoral motor and saphenous sensory NCS and needle EMG, NCE stimulation charge thresholds, and tetanic knee moments, before and after initiating an exercise program with stimulation. We report the electrophysiological and clinical outcomes of the first-in-man deployment of bilateral 8-contact C-FINEs on the proximal femoral nerve trunks of one subject with SCI (Fig. 2).

## Methods

**Fig. 3:**
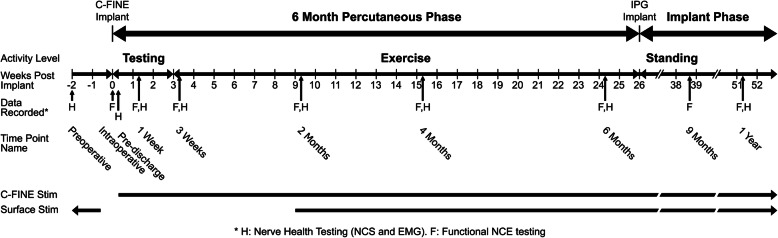
Experimental timeline. “Nerve health testing,” including NCS and needle EMG, was performed at 1 time-point preoperatively and 7 time-points postoperatively. “Functional testing,” including charge threshold and moment measurements, was performed intraoperatively without moment measurements and at 7 time-points postoperatively. Moment was collected at all postoperative time-points but was restricted to twitch moments only until the third week postoperatively. The percutaneous phase ended when a 16-channel IPG, full standing neuroprosthesis system was implanted. This required a reduction in the number of contacts for knee extension from 16 to 6 across both C-FINEs to allow stimulus channels to be assigned to intramuscular electrodes for hip and trunk extension

A combination of electrodiagnostics and metrics of neuroprosthesis functional performance was employed to quantify femoral nerve health and nerve-cuff interactions at seven time-points over a 1-year period postoperatively (Fig. 3). Measures of neuroprosthesis performance included stimulation charge thresholds, tetanic knee extension moments, and measurement of weight supported through the subject’s legs while standing. NCS and needle EMG systematically compared neuromuscular function to preoperative baseline levels. For the first 6 months postoperatively, percutaneous leads allowed a custom stimulator, the Universal External Control Unit, UECU, (Case Western Reserve University, CWRU, Cleveland OH) [49] to access to all 16 contacts across bilateral C-FINEs (CWRU, Cleveland OH). After this period, percutaneous leads were removed and 3 contacts per C-FINE selective for isolated knee extension were connected to an implanted pulse generator (IPG) that delivered stimulation through these 6 contacts. The IPG in this study was the 16-channel implanted stimulator-telemeter (IST-16) developed at CWRU and the Cleveland Department of Veterans Affairs [50–52]. Electrodiagnostic studies interrogated the whole nerve either via surface stimulation delivered via a clinical Alpine Keypoint EMG Unit (Alpine Biomed ApS, Skovlunde Denmark) or via the C-FINEs when surface stimulation was impractical.

### C-FINE Implantation

The C-FINE (Fig. 1a) is a NCE designed to match the naturally oblong cross section of many peripheral nerves [38, 53] and gently reshape them into a flatter cross section. A flattened architecture allows for isolated stimulation of functionally distinct fascicles and the placement of more stimulating contacts around a nerve [36]. The C-FINE employs graduated stiffness to reshape the nerve along the middle of the C-FINE while remaining highly flexible along the length of the nerve to accommodate nerve bending [43] (Fig. 1a, Top). The C-FINEs had 4 platinum-iridium contacts with an exposed surface area of 0.5 mm^2^ evenly distributed on each of the top and bottom interior surfaces of the electrode for a total of 8 contacts.

We implanted 8-contact C-FINEs bilaterally on the proximal femoral nerves of a male volunteer (age 25, 5 years post-injury) with SCI (C5 American Spinal Injury Association impairment scale C). Inclusion in this study required a subject who has little to no volitional control of muscles which would assist in standing. At preoperative baseline, the C-FINE volunteer had limited volitional control of his lower extremity muscles. Medical Research Council (MRC) manual muscle testing [54] was conducted at several visits. Preoperatively, this testing showed the following: volitional hip flexion was 3/5 (strength against gravity) on the right, and was 2/5 (strength parallel to gravity) on the left; and knee extension was 2/5 bilaterally. These results were consistent with the inclusion criteria.

Lower density (i.e. 4-contact) NCEs do not have the resolution to separately activate distinct functional groups of fascicles on the common femoral nerve trunk proximal to branches to both knee extensors and hip flexors [55]. The anticipated selectivity of 8-contact C-FINEs [37] justified implanting the devices at this location. Because of the potential of the femoral nerve at this site to move and bend beneath the inguinal ligament during hip flexion [39–42], a surgeon verified that the C-FINEs did not get compressed or pulled underneath the ligament with each hip flexed to near 90°. A redundant loop for strain relief (Fig. 1b) was left in the distal leads close to the C-FINEs prior to subcutaneous tunneling to proximal connector sites in the abdomen. The strain relief loop reduced the potential for transmitting tethering forces to the devices or nerves during hip movement.

Intraoperatively, an external stimulator, the UECU elicited contractions via stimulation through the C-FINE contacts. Monopolar EMG needles inserted into muscles innervated by the femoral nerve (Fig. 1d), recorded the evoked EMG intraoperatively to calculate stimulation charge thresholds and motor nerve conduction velocities (MNCVs). Following EMG recording, the leads from the C-FINEs were first passed to abdominal spring-and-pin connectors [56]. Helical wound percutaneous leads [57, 58] connected to each C-FINE allowed access to all 8 individual contacts. These percutaneous leads were then tunneled subcutaneously and individually to exit sites on the proximal anterolateral thighs **(**Fig. 2a). The subject cleaned, monitored, and re-dressed the percutaneous interfaces for the next 6 months, during which time he experienced only minor complications such as an occasional mild erythema (white arrow in Fig. 1c).

Intraoperatively and 3 weeks post-implantation, stimulation with single pulses of stimulation avoided tetanic contractions and minimized risk of NCE movement. Following this 3-week period, we initiated exercise paradigms with tetanic stimulation. Following the 6-month percutaneous phase, the subject received a fully implanted standing neuroprosthesis (Fig. 3). Percutaneous leads were removed and 6 out of 16 C-FINE contacts for isolated knee extension bilaterally were connected to an IPG, along with intramuscular electrodes for hip and trunk extension bilaterally, including bilateral quadratus lumborum, erector spinae, gluteus maximus, and posterior portion of adductor magnus and unilateral hamstring (right) and gluteus medius (left) (the “Intramuscular Electrodes” shown in Fig. 2a).

### Electrodiagnostic testing

**Fig. 4:**
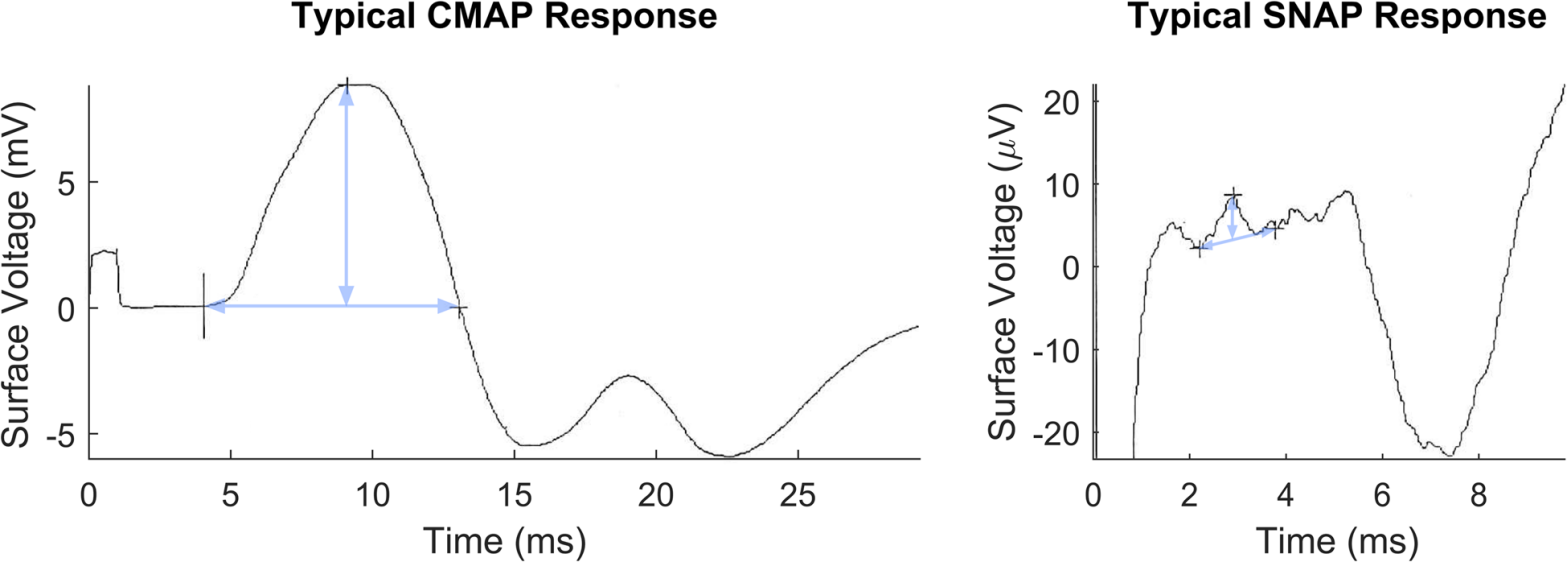
Example of typical CMAP and SNAP. CMAP (left) of RF after surface stimulation of femoral nerve. Blue arrows indicate duration (horizontal) and amplitude (vertical) of the CMAP which gave an amplitude of 8.8 mV, and area of 47.8 mV ms. SNAP of the saphenous nerve (right) was recorded after stimulation of sensory fibers. The stimulus artifact extended to roughly − 40 μV on this plot but was cropped for readability. Blue arrows again measure duration and amplitude and this SNAP had an amplitude of 4.2 μV

Before implantation and throughout the 1-year follow up period, a neurologist subspecializing in electrodiagnostics and neuromuscular diseases conducted all electrodiagnostic testing. Two weeks prior to implantation, NCS and needle EMG studies evaluated baseline nerve health. Preoperative NCS of bilateral lower extremities quantified saphenous, sural, and superficial peroneal sensory nerve action potentials (SNAPs), and peroneal, tibial, and femoral CMAPs. Examples of typical rectus femoris (RF) CMAPs and saphenous nerve SNAPs are shown in Fig. 4 along with illustration of calculation of their amplitude and area. Needle EMG was performed on 9 muscles per leg innervated by these nerves. EMG was interpreted by an experienced electromyographer according to clinical standards [59, 60]. Spontaneous activity, such as fibrillation potentials, was scored on an ordinal scale to indicate the frequency and proportion of areas showing these discharges. Pre- and post-operative testing focused on NCS of the femoral and saphenous nerves, and needle EMG of femoral nerve innervated muscles, including vastus medialis (VM), vastus lateralis (VL), RF, and sartorius (Sart). Vastus intermedius (VI) was not included because it is not routinely included in routine clinical needle EMG testing, and we aimed to apply methods that were consistent with established clinical practices in this study for future reproducibility.

Saphenous SNAPs were elicited with surface stimulation of sensory fibers on the medial calf, between medial gastrocnemius and the tibia, 14 cm proximal to recording electrodes. The SNAP was recorded with bar electrodes with the active electrode midway between the tibialis anterior tendon and the medial malleolus and reference electrode positioned distally [61, 62]. For the purposes of this study where the NCEs were placed on the femoral nerves, postsurgical testing focused on the femoral motor nerve responses, and its terminal nerve branch, the saphenous sensory nerve. It was helpful to track the saphenous studies, since sensory responses are three orders of magnitude smaller than motor responses, and hence more susceptible to axonal or demyelinative nerve damage [63].

CMAPs were elicited either with surface stimulation (“surface-elicited”) of the femoral nerve at the groin using standard commercially available electrodiagnostic test equipment (Alpine Keypoint) or via stimulation through the C-FINEs (“C-FINE-elicited”) via the custom stimulator (the UECU initially and then the IST-16 once implanted). Responses were always recorded at RF with surface electrodes using clinical EMG equipment (Alpine Keypoint). The surface stimulation and recording represent the clinical standard of care and are generally preferred to replicate clinical practice. However, measurements made while stimulating through the C-FINEs allowed additional testing, including CMAP measurements made with the subject sitting (hip flexed to 90°) compared to supine to evaluate for potential femoral nerve compression during hip flexion. Surface stimulation of the femoral nerve with hips flexed is impractical since the groin is blocked. Additionally, surface stimulation over the surgical site was contraindicated in the perioperative period (through the 3-week time point) making stimulation via the C-FINEs necessary during this time period.

Three weeks postoperatively, custom pre-programmed stimulation paradigms [12, 64] were initiated to recondition the knee extensor musculature. The exercise paradigm generated contractions of quadriceps muscles to build knee extension strength with high load and low repetition exercises with progressively increasing ankle weights while sitting. To build endurance, the stimulation paradigms utilized long duration and low load exercises. These exercise programs continued throughout the 6-month percutaneous phase. After 6 months, the standing phase began with implantation of the IPG [50, 51]. The subject continued to build knee extension strength and endurance with a take-home standing rehabilitation program, which he began to use approximately 6 weeks after IPG installation (8 months after C-FINE implantation).

### EMG evaluating Neuroprosthesis function

Intraoperatively and at all postoperative time-points, pairs of monopolar EMG needles recorded the responses to stimulation delivered through the C-FINEs. These determined stimulation thresholds, saturations, and functional responses. During the 6-month percutaneous period, the externalized leads to the C-FIINE contacts were connected to a custom-designed current-controlled stimulator (UECU) that delivered monopolar, charge-balanced, biphasic pulses with pulse amplitude ranging from 0.1 to 2.0 mA and pulse widths ranging from 1 to 255 μs. A surface return electrode was placed over the left lower quadrant of the abdomen, which was chosen to best replicate the future site of the IPG. When the IPG was implanted, the titanium case was sutured to the abdominal fascia and acted as the return electrode. Pulse amplitude was limited so that charge densities per phase remained under the suggested level for electrical stimulation given by:

1 $$ \log (QD)=k-\log (Q) $$

where QD is the charge density per phase (μC/cm^2^) and Q is charge per phase (μC) [65]. Based on prior work, k < 1.5 is considered safe for continuous pulse trains up to frequency of 50 Hz [66–68]. We limited pulse amplitude so that k was less than 1.5 for all injected charges. In most cases, pulse amplitudes were 0.8 mA and occasionally up to 1.4 mA. “Twitch stimulation” involved stimulating with a single pulse with at least 1 s between pulses. “Tetanic stimulation” delivered a train of pulses at 20 Hz.

Prior to stimulation, a surface reference electrode (2″ × 4″, Nicolet-VIASYS, Madison, WI) was placed over the greater trochanter of the contralateral leg. R.A. placed monopolar EMG needle electrodes (Nicolet 27 to 28 gauge needles, 13 mm – 50 mm length (Natus Medical Inc., Pleasanton, CA) approximately 2 cm apart into knee and hip extensor muscles innervated by the femoral nerve (Fig. 1d). EMG was recorded independently and simultaneously from the three heads of the vasti (VM, VI, VL), RF, and Sart.

Stimulation delivered through each contact on the C-FINEs at all measurement intervals elicited twitch EMG responses. Each pair of EMG needle electrodes was AC-coupled to a differential amplifier (B&L Engineering, Tustin, CA) with passband 12–2975 Hz and gain 325. Programmable amplifiers (1902, Cambridge Electronic Design, CED, Cambridge, UK) further low-pass filtered at 1 kHz and amplified the signal for a total gain of 1155. An A/D DAQ board (BNC-6259, National Instruments, Webster, TX) sampled the signal at 2.5 kHz and custom MATLAB (MathWorks, Natick, Massachusetts) code interfaced with the programmable amplifiers and the UECU. EMG responses of each muscle were rectified, integrated over the duration of the M-wave (3 ms to up to 30 ms after stimulation), and normalized by the maximum twitch for the muscle following supramaximal stimulation on all contacts [69] at either 0.8 or 1.4 mA and 255 μs. These EMG twitches were repeated 3 times at every stimulation parameter to provide a mean and standard deviation for each stimulation input. The distance between each muscle and the C-FINE was approximated by measuring the distance from surgical scar to EMG needles over the surface of the thigh. Dividing the distance between the C-FINE and the recording needles for each muscle by the onset latency of the CMAP for that muscle determined MNCVs. Onset latency was defined as the time between stimulation and M-wave onset, with a 0.5 ms offset to approximate neuromuscular junction delay [70, 71]. The MNCVs averaged across all of the muscles provided the conduction velocity for the femoral nerve.

Calculation of the stimulation threshold charges follows:

2 $$ {Q}_{\theta }=I\ x\ {PW}_{\theta } $$

The current, *I* (either 0.8 mA or 1.4 mA) applied through each contact was the minimum current able to reach supramaximal stimulation by the maximum pulse width of 255 μs. Thus, supramaximal contractions were elicited either with a pulse amplitude of 0.8 or 1.4 mA or at a pulse width of 255 μs. Current was adjusted on a contact-by-contact basis, but remained consistent for each contact across all time-points to eliminate a potential source of variability in charge threshold calculations [72, 73]. *PW*_*Ɵ*_ was defined as the pulse width at which EMG response reached 10% of its supramaximal stimulation value. Charge threshold stability based on EMG was tracked for all 16 contacts during the 6 month percutaneous phase, and then derived from the moment measurements for the 6 contacts connected to the IPG at 9 months and 1 year postoperatively.

### Surrogate measures of muscle strength

Starting at 3 weeks postoperatively, and for all remaining time-points, a 6 degree-of-freedom load cell (JR3 Inc., Woodland, CA) measured isometric, tetanic knee extension moments via software written in LabVIEW (National Instruments, Austin, TX) and MATLAB with the knee fixed at 20° flexion and with the distal thigh fixed to the seat of a robotic dynamometer (Biodex, Shirley, NY). The load cell was employed, rather than the dynamometer measurement head, for its superior sensitivity and noise characteristics which were particularly important for recruitment properties close to threshold which are critical to assessing selectivity. The dynamometer head was aligned with the center of rotation of the knee so the torque measured at the load cell matched that at the knee. Moment data were low passed filtered at 31.25 Hz, sampled at 150 Hz, normalized by the subject’s body mass (54.5 kg initially) and reported in Nm/kg.

Tetanic stimulation was delivered through each contact at 20 Hz for approximately 3 s with at least 15 s of rest between repeated bursts to avoid fatigue, as described for previous standing neuroprosthesis studies [26]. We determined whether there was a significant linear relationship between knee moment and time-point in the study by fitting a linear regression to the yearlong mean moment data for each leg and using a t-test on the null hypothesis that slope of the fitted line is 0, which would indicate no change as a result of stimulated exercise.

As a secondary measure of quadriceps strength and reconditioning, thigh circumference was measured at all postoperative time-points and compared to a pre-exercise baseline. The same individual consistently measured circumference at a site 15 cm proximal to the site of attachment of the quadriceps tendon to the patella. By assuming the thigh was approximately circular in cross section, a squared ratio of circumferences provided an estimated ratio of areas.

To be functionally relevant, stimulation must generate moments sufficient and stable enough to support a subject’s body weight through the feet. Starting approximately 7 months postoperatively, standing with stimulation was added to the exercise and rehabilitation program. The subject positioned his feet on a set of force plates (AMTI, Watertown MA) to measure right and left ground reactions while he maintained quiet standing with his heuristically tuned take-home “standing” stimulation pattern. He used a walker that was wider than the force plates to assist with balance so that only the body weight transmitted to the ground through his feet were recorded. The vertical components of the ground reaction forces at his feet were normalized by his body mass to calculate percentage of body weight supported by the lower extremities.

## Results

Preoperative needle EMG showed limited activation with otherwise normal motor unit potential morphology and recruitment patterns in the bilateral tensor fascia lata and right flexor digitorum longus muscles. No other tested muscle in the bilateral lower extremities showed volitional activity: Sart, RF, VL, VM, tibialis anterior, medial gastrocnemius, nor extensor hallucis longus. Preoperatively, no tested muscle revealed electrophysiologic evidence for active or chronic denervation. At baseline, sensation was decreased (50% of normal) to light touch and absent to pinprick throughout the lumbosacral dermatomes, including the saphenous nerve distribution.

Preoperative NCS in the lower extremities revealed essentially normal bilateral sensory nerve (saphenous, superficial peroneal and sural) and motor nerve (femoral, peroneal and tibial) responses. The left saphenous sensory response showed borderline decreased amplitude, but was otherwise normal. Preoperative NCS and EMG were consistent with a central etiology, such as SCI, for his clinical weakness.

### Motor nerve conduction studies

**Fig. 5:**
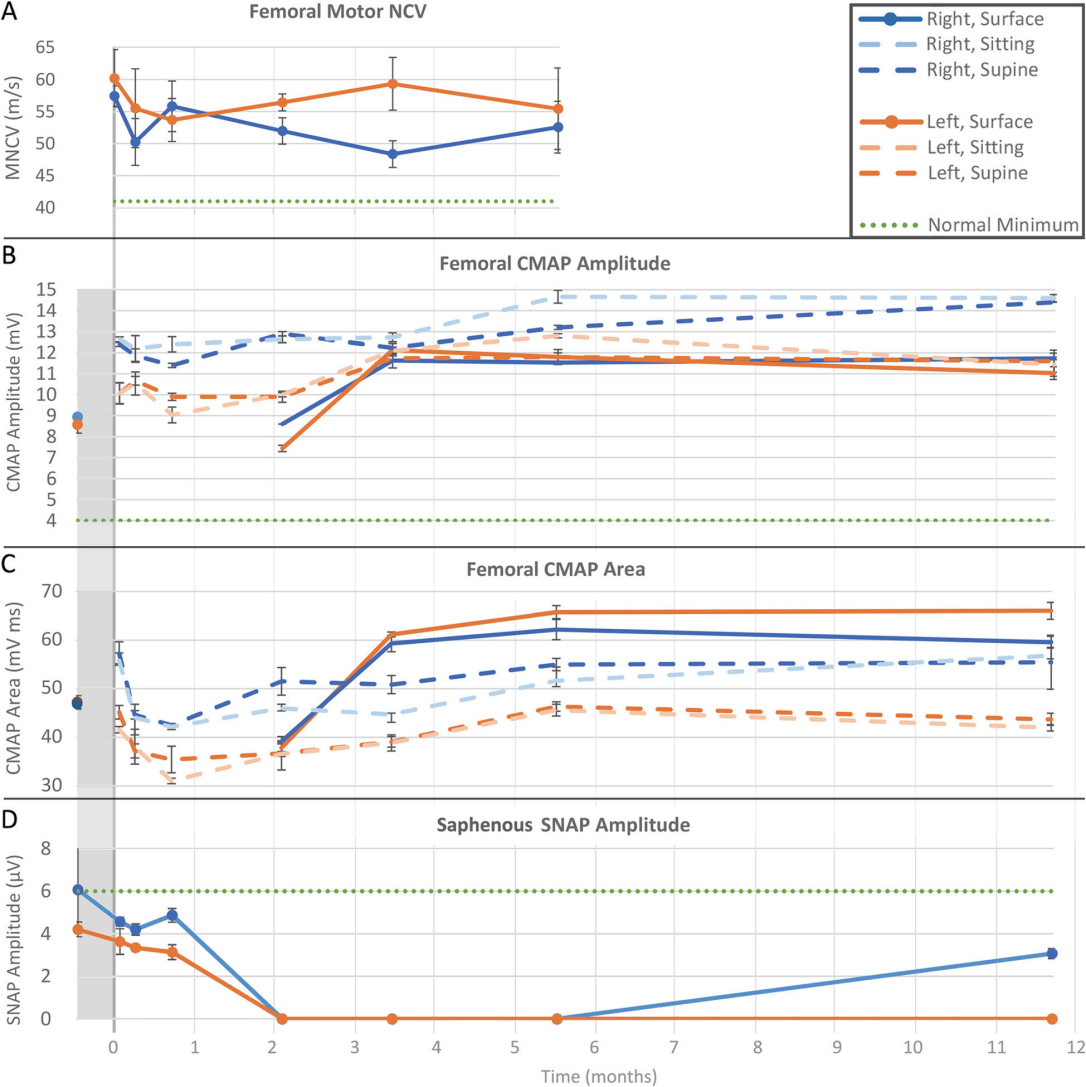
Summary of femoral motor and saphenous sensory nerve conduction studies. Time axis is the same for all plots with preoperative period shaded. Measurements in this region are used as baseline values. Dashed lines indicate C-FINE-elicited CMAPs. Solid lines indicate surface-elicited CMAPs (the clinical standard). CMAP is measured at RF. **a** Femoral MNCV. Mean MNCV of quadriceps muscles compared to clinical minimum normal velocities. Error bars represent SEM between velocities to the heads of quadriceps muscles. **b** Femoral CMAP Amplitudes: C-FINE-elicited compared to baselines surface-elicited 2 weeks preoperatively and minimum normal values. C-FINE-Elicited CMAPs also compared with subject sitting (hips flexed 90°) and supine. Error bars represent standard deviations between 3 trials of supramaximal stimulation. **c** Femoral CMAP Areas collected and presented identically to CMAP amplitudes and at the same time points. **d** Saphenous SNAP Amplitudes. Postoperative amplitudes compared to baseline elicited 2 weeks preoperatively and minimum normal amplitudes

C-FINE stimulation with recording at all heads of the quadriceps revealed consistent, symmetric, and normal MNCV above 41 m/s (one sample t-test *p* < 0.05 that the average MNCV > 41) throughout the 24-week percutaneous phase of the study (Fig. 5a). Although there was an apparent decrease in MNCV from intraoperative measurements compared to 1 week postoperatively, this drop was not statistically significant (*p* = 0.3 on the left and *p* = 0.07 on the right). There was no statistically significant difference between the MNCVs on the right as compared to the left (*p* = 0.06). Femoral nerve latencies elicited by surface stimulation at the inguinal ligament and measured at the RF were recorded preoperatively and again at 2, 4, 6, and 12 months. These were always within normal limits (< 6 ms).

Both surface-elicited (solid lines in Fig. 5b and c) and C-FINE-elicited (dashed lines) CMAPs showed increasing amplitudes and areas over the course of this 1-year. The increase in CMAP amplitude and area occurred after the subject started his rehabilitation exercise program 3 weeks postoperatively. Surface-Elicited-CMAP amplitudes and areas were above baseline at all time-points except one, 2 months postoperatively, when amplitudes averaged 9.6% and areas 18.2% below baseline bilaterally. This time-point correlated with an increase in the stimulation threshold (discussed below), which limited the ability for supramaximal stimulation via surface stimulation. In contrast, C-FINE-elicited CMAP amplitudes at 2 months essentially equaled (+ 1.5%) baseline CMAPs. This indicates that the dip in surface-elicited CMAPs at 2 months may be due to increased subcutaneous cellularity or other difficulty achieving a full stimulated contraction percutaneously. By 6 months postoperatively, with regular exercise, surface-elicited CMAP amplitudes and areas increased by an average of 31 and 34%, respectively. From 6 months to 1 year postoperatively, all CMAP values were stable and essentially unchanged by the standing exercises initiated around 8 months postoperatively. There was no significant decrease (paired t-test, *p* = 0.06 on the right and *p* = 0.85 on the left) in the C-FINE elicited CMAP amplitudes secondary to hip flexion.

### Sensory nerve conduction studies

Saphenous SNAP amplitudes were normal in the right lower extremity, and borderline normal in the left lower extremity. Bilateral saphenous sensory responses were stable at 3 weeks postoperatively, but absent by 2 months (Fig. 5d). The right saphenous SNAP started to recover by the 1 year test date; however, the left saphenous response remained absent until this time-point. Subjectively, the subject did not notice any clinical change in his lower extremity sensation; however, his sensation was already compromised in the setting of his SCI. When obtained, saphenous SNAPs demonstrated velocities greater than 41 m/s, ranging from 42 to 51 m/s. Return of right saphenous SNAP by the 1 year test date suggests a regeneration rate of roughly 2–3 mm/day from the time of surgical manipulation which is consistent with other studies for distal peripheral nerve axonal regeneration [74, 75].

### Needle EMG

Preoperatively, needle EMG showed normal insertional activity, without any spontaneous activity. At 1, 3, and 9 weeks postoperatively, rare fibrillation potentials were seen in the following muscles: Sart and VM on the right; and Sart, RF, and VL on the left (Table 1). As expected, more proximal muscles (Sart, RF) showed signs of active denervation initially, and as the proximal muscles improved, the distal ones showed rare fibrillation potentials. No fibrillation potentials were seen at or after 15 weeks postoperatively, indicating that nerve irritation, inflammation, or damage resolved after the intraoperative or perioperative periods.

**Table 1:**
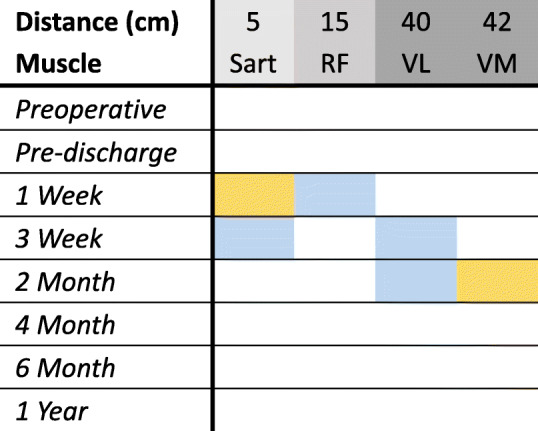
Summary of locations and time-points of active denervation. Active denervation never exceeded rare fibrillation potentials at any time-point or location. First column indicates distance from C-FINEs to where EMG needle typically inserted in muscle. Yellow (right leg) and blue (left leg) boxes indicate rare fibrillation potentials at a specific time-point. Sart, sartorius; RF, rectus femoris; VL, vastus lateralis; VM, vastus medialis

### Stimulation charge thresholds

**Fig. 6:**
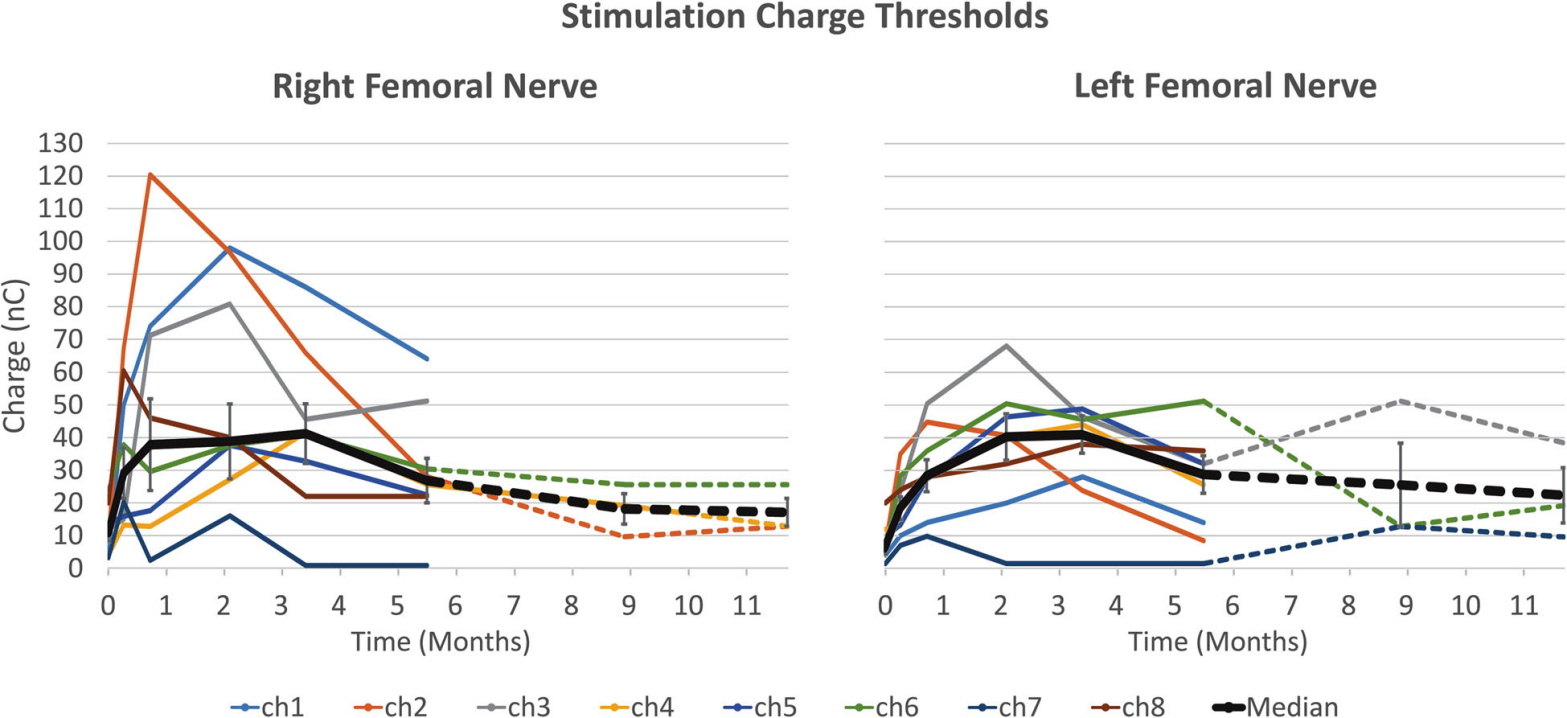
Stimulation charge thresholds from implantation through 1 year postoperatively. Thresholds defined by charge when rectified, integrated, normalized EMG (solid lines) or moments (dashed lines) reach 10% of maximum recorded value for a given muscle. Error bars on median measure represent SEM. Reduction in data points after 6 months due to removal of percutaneous leads and 3 out of 8 contacts chosen per C-FINE and connected to the IPG

Stimulation charge thresholds were calculated for each available C-FINE channel at all time-points (Fig. 6). Fifteen out of 16 contacts had charge threshold below 100 nC at every time-point and the remaining contact’s charge threshold only exceeded 100 nC for a single measurement. The mean of the charge thresholds was lowest at implantation at 9.7 nC ± 1.7 (SEM) over all contacts. These rose postoperatively, and peaked around 2 months at 45.8 nC ± 6.9 (SEM). At the end of the 6-month percutaneous phase, thresholds across all 16 contacts averaged 27.8 nC ± 4.4 (SEM). At 6 months the mean threshold across the 6 contacts selected to be connected to the IPG was 28.1 nC ± 9.2 (SEM), and at 1 year it was 19.7 nC ± 6.2 (SEM), revealing that thresholds continued to decrease as expected. Most of this drop occurred by 9 months after which values appeared to plateau. By 1 year, the thresholds appeared to be stable and were not expected to decline further [2]. These charge thresholds and the time course of their changes are consistent with previous NCE studies [2, 26].

### Surrogate markers for muscle strength

**Fig. 7:**
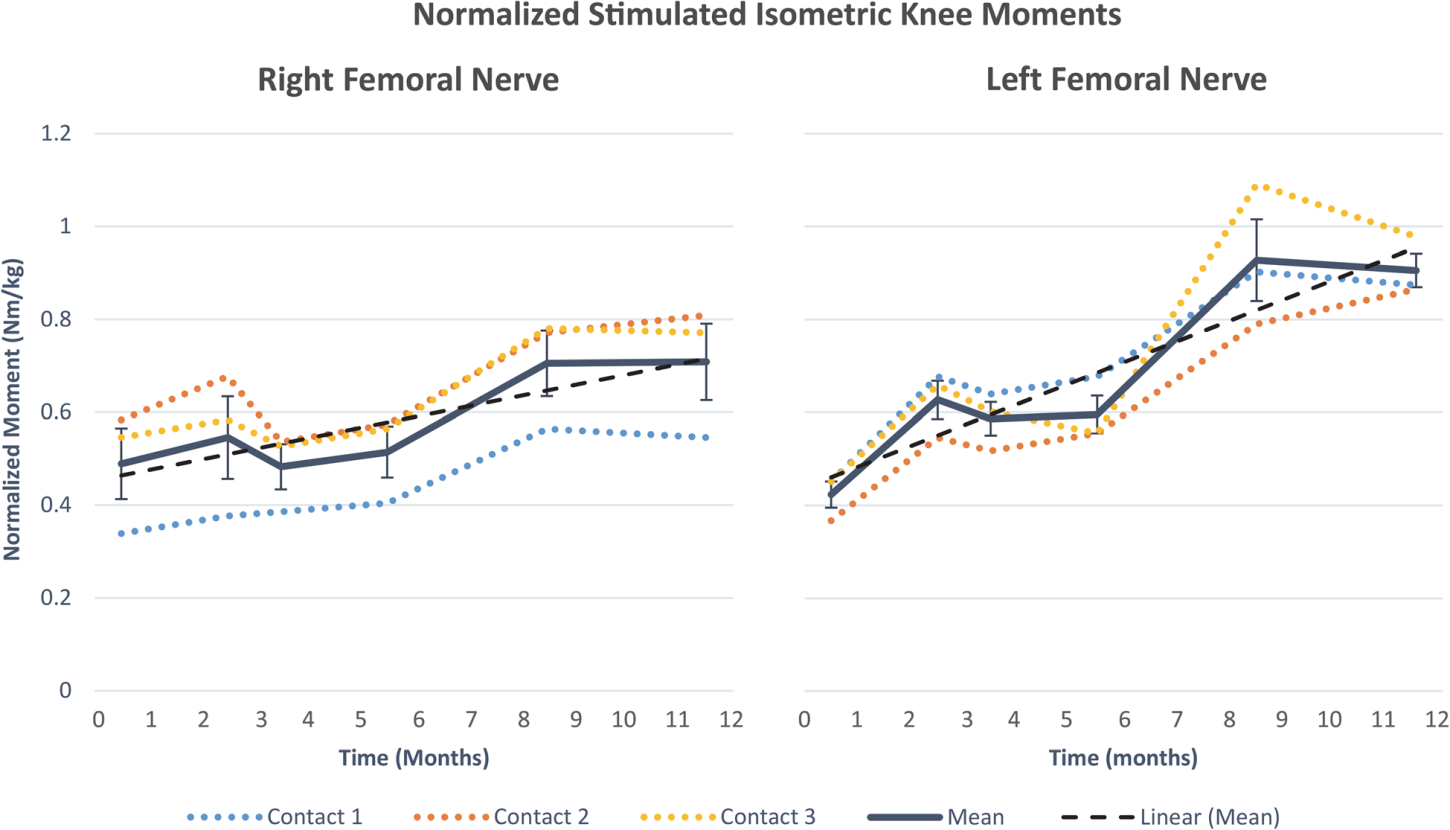
Knee moments normalized by body mass over 1 year on each of the selected knee extensor selective C-FINE contacts at 0.8 mA, 255 μs. These contacts were chosen because they were able to achieve strong knee extension moments with insignificant hip flexion. Error bars indicate standard deviation between contacts. Dashed line represents linear best-fit to mean tetanic moments. Based on linear regression t-testing, an increase in moment over 1 year is significant (*p* < 0.01 on the left and *p* < 0.02 on the right)

From the initiation of tetanic stimulation through 1 year of neuroprosthesis use for exercise and standing, maximum single-contact tetanic knee extension moment for the 6 contacts connected to the IPG increased by an average of 79% ± 16 (SEM) (Fig. 7). Linear regression t-tests demonstrated a significant increase in moment during this study on both the left (*p* < 0.01) and right (*p* < 0.02) legs. The initial average moment of 0.42 Nm/kg per contact on the left increased by 110% to produce an average moment of 0.89 Nm/kg per contact. The initial average moment of 0.49 Nm/kg per contact on the right increased by 44% to produce an average moment of 0.70 Nm/kg per contact. Despite these increases in stimulated knee moments, repeated manual muscle testing did not indicate any consistent nor functional significant change in volitional knee moment.

Over the first 5 months of stimulated exercise, thigh circumference increased by 11 and 12% (from 31.0 to 34.4 cm and from 30.5 to 34.3 cm) on the right and left sides, respectively. With the addition of stimulated standing exercise in the second phase of this trial, thigh circumference increased an additional 6% (to 36.5 cm) on the right and 8% (to 37.0 cm) on the left by 1 year. This resulted in total circumference gains of 18 and 21% on the right and left thighs, respectively. Thigh cross-sectional areas derived from these circumference measurements, increased by 39% on the right and 47% on the left, suggesting increases in force generating capacity with reconditioning exercise and standing [76, 77].

**Fig. 8:**
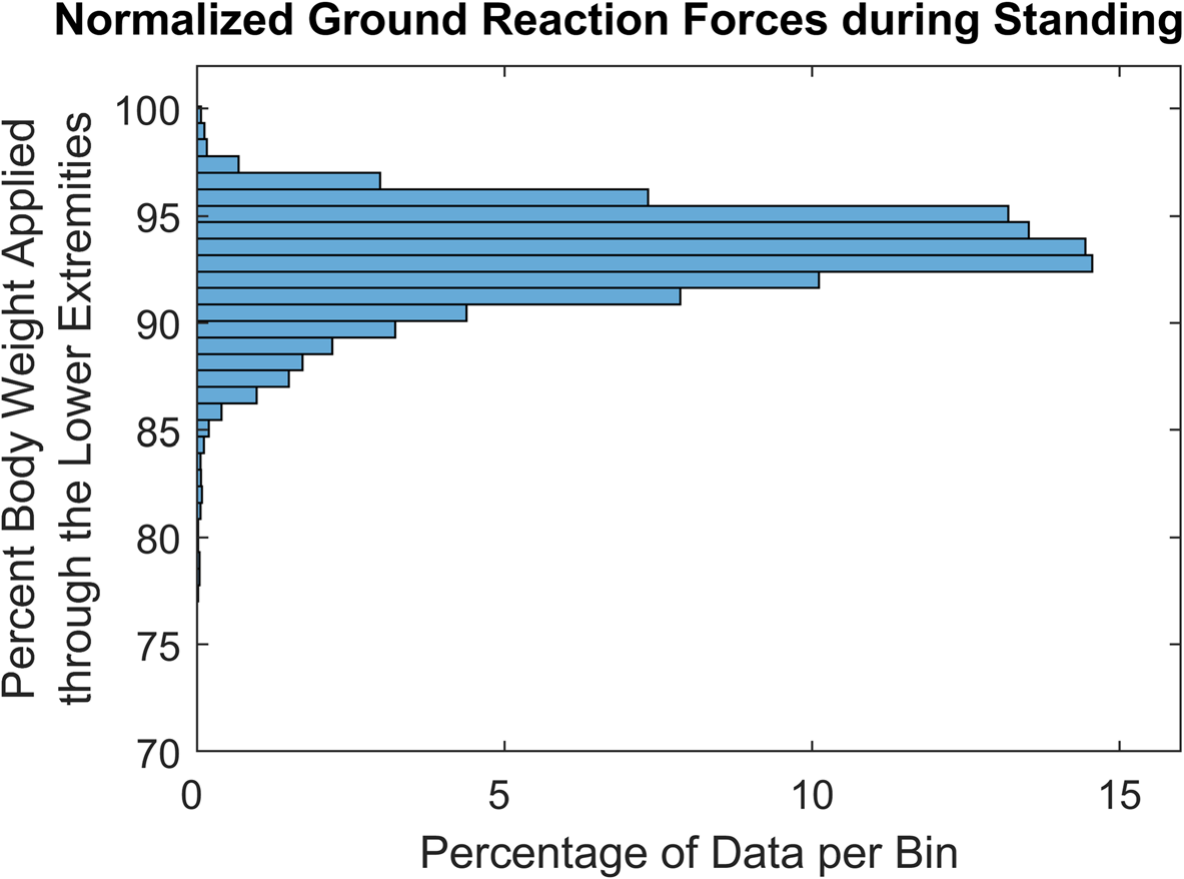
Histogram of summed and normalized vertical component of ground reaction forces measured during quiet standing with take-home “standing” pattern, indicating percentage of body weight supported through legs

Approximately 7 months postoperatively, the subject began standing with his take-home “standing” patterns of stimulation. Force plate data from roughly 5 min of quiet standing showed that 93.1 ± 2.1% of his body weight was supported by his legs while standing (Fig. 8).

## Discussion

This study reports the neurophysiological results secondary to implantation of C-FINEs on bilateral femoral nerves as part of a standing neuroprosthesis in a subject with cervical SCI. While a larger number of NCEs across more subjects and different nerve sites will help bolster these results in the future, there are several important implications from this study. Our findings represent baseline electrodiagnostic measures and changes that can be reasonably expected over 1 year of implantation and chronic use of C-FINEs on peripheral nerves.

There are multiple possible etiologies for the mild electrodiagnostic changes seen on EMG testing. Nerve manipulation during dissection and NCE placement can cause neurapraxia or mild axonotmesis. C-FINE movement along the nerve in the perioperative period, prior to stabilization with encapsulation tissue, could also cause nerve irritation. Fibrillation potentials seen with needle EMG started approximately 1 week after surgery and cleared between 9 to 15 weeks postoperatively. This time course is consistent with neurapraxia or mild axonotmesis occurring during NCE implantation, revealing that there was not on-going denervation or irritation.

The surface-elicited CMAP amplitudes and areas decreased by roughly 20%, at 2 months (Fig. 5b and c solid lines). However, C-FINE-elicited CMAPs (dashed lines) were similar to baseline at the 1–2-month timeframe. This discrepancy is associated with a significant increase in the stimulation charge threshold at this point. The elevated threshold may have limited the ability to reach a supramaximal response, especially through surface stimulation. Increases in stimulation charge thresholds in the first several months postoperatively is often attributed to inflammation between electrode and nerve and is well established [2, 26, 30]. A similar increase in charge thresholds was observed for stimulation through the C-FINEs in this study, which rose almost five-fold over the first 2 months postoperatively. However, over the last 10 months of this study, thresholds consistently declined and stabilized as expected.

NCS also revealed saphenous SNAP loss and partial recovery between 6 months to 1 year. Combined with the presence of temporary mild fibrillation potentials, these findings are consistent with mild axonal loss and regeneration [45, 78]. The rate of recovery for the right saphenous SNAP is consistent with established axonal regeneration rates [45, 46] and support temporary, perioperative irritation. Postsurgical neurapraxia tends to have a positive prognosis [79] and these results show a positive functional outcome in this subject. Moreover, the subject reported no appreciable changes in his 50% preserved sensation over the saphenous dermatome at any time. All other studies indicated that the C-FINEs had a positive impact on the rehabilitation potential for the muscles innervated by the femoral nerves, and improved functional status. We recommend that electrodiagnostic studies be used to track neurophysiological changes due to an implanted NCE, since this data will allow for clinical determinations about NCE viability and nerve health.

Despite mild changes in neurophysiology, the standing neuroprosthesis deployed in this study was clinically successful. After 3 months of training, this subject was able to stand in excess of 30 min (Figs. 2 and 8), after being wheelchair dependent for the previous 5 years. These standing times may be attributable to the performance of the C-FINEs, although it should be noted that standing times produced by neuroprostheses are extremely variable and dependent on a multitude of factors [1, 80]. Each of the three individual contacts within each C-FINE generated four times the 0.135 Nm/kg of knee extension [81–83] required for quiet standing, and each C-FINE generated twice the 0.4 Nm/kg [83, 84] required for the sit-to-stand transition. In fact, biomechanical measurements indicated that all of his body weight, excluding his arms [85, 86], was supported by his legs while standing (Fig. 8). Although these knee moments and standing times may be influenced by the subject’s relatively low body mass, adherence to the exercise and rehabilitation protocol, or other factors, the performance of the nerve-based electrodes made a considerable contribution to the functional outcome. A deeper discussion of the functional efficacy of the C-FINEs and their selectivity over time can be found in another study [87] where we detailed the ability of the C-FINEs to separate knee extension from hip flexion, independently activate separate populations of knee extensor motor units, and document recruitment patterns and selectivity changes over time—intraoperatively, perioperatively, and chronically. This increased selectivity can be used to recruit functionally disparate groups of muscles, as well as to use synergistic muscles to delay fatigue [88] or increase the dynamic range of force generation.

After 1 year of various quadriceps focused exercises, notable increases or improvements were noted in muscle bulk, strength generated with stimulation, and electrodiagnostic measures. Bilaterally CMAPs increased an average of 31%, and isometric moments increased an average of 79%. Amplitudes and areas of femoral CMAPs increased during the initial 6 months of exercise with stimulation through the NCE. However, there was no appreciable increase from 6 months to 1 year, despite significant increases in elicited knee extension moment and thigh circumference indicating muscle hypertrophy. Notably, CMAP amplitudes, areas, and duration remained unchanged with hip flexion, indicating that the C-FINE was flexible enough to accommodate nerve bending in proximity to the inguinal ligament.

This study utilized clinically-standard semiquantitative interpretation of needle EMG activity, in particular with regard to classifying the amount of spontaneous activity. Following these standards for EMG interpretation allow an experienced electromyographer to quickly record from several muscles, lowering the barrier for this type of testing. However, they require the time of an experienced electromyographer and may not capture the full variability in responses across the sampled muscles. For instance, the standard deviation calculated on twitch CMAPs (Fig. 5b and c) were calculated from three supramaximal twitches during a single testing session. This does not capture the possible variability expected from day to day, at different extremity temperatures [89], or across variations in recording electrode placement. Additionally, three samples affords only two degrees of freedom which increases the range of a confidence interval in a t-distribution roughly 2–4 fold compared to higher degrees of freedom [90]. Future studies may benefit by exploring other quantitative EMG methods [59, 78] or expanding the number of stimuli performed at each parameter set [91].

Limitations of this study included session-to-session variability in isometric moment measurements, which were attributed to several factors. First, there was inconsistency with day-to-day differences in muscle fatigue. This unpredictability is exaggerated in individuals with SCI, especially during stimulation induced contractions of the paralyzed muscles [92]. Additionally, there may be methodological or technical sources of variability, such as the uncertainty repeated positioning of the knee during dynamometer measurements of knee moment. Isometric knee extension moments were measured at 20° of knee flexion to reduce the risk of injury during tetanic contractions with the knee flexed due to the length-tension properties and moment arm of the quadriceps. At this angle isometric knee extension moments are highly sensitive to changes in knee angle. At 20° of knee flexion, moment is about 38% of maximum (occurring around 80° knee flexion). If the knee is flexed to 30°, moment rises to roughly 51% of maximum, and if the knee is extended to 10°, the moment falls to 29% of maximum [93]. Thus, changes in 10° from the target knee angle of 20° of flexion can result in a rise of 34% or drop of 23%. Despite the multiple sources of variability, increase in knee moment during the study were statistically significant (*p* < 0.01) and averaged 79% across both legs. These gains are large enough to be clinically significant as well. Although not statistically significant, the moment gains were much larger in the left leg (110%) than the right (44%). At baseline, there was more atrophy and weakness in his left lower extremity, possibly due to asymmetric disuse and deconditioning resulting in him favoring his right leg. The C-FINE stimulation and rehabilitation paradigms may have compensated for the asymmetry and allowed him to use both his legs equally, so that by the end of the study weight bearing on his legs during standing with the neuroprosthesis was more symmetric. Another limitation is the potential variability in calculating MNCV as we have here. The ideal measurement would be along a known and repeatable portion of the nerve with stimulation at the same two locations. Because we did not have access to the nerve itself after electrode implantation, we had to approximate distance from C-FINE to EMG needles. Additionally, we had to use data from the literature to approximate the delay at the neuromuscular junction. These factors may have made our MNCV measurements less accurate than they otherwise could be.

Another limitation is that all 16 contacts of the C-FINEs were only accessible during the 6-month percutaneous phase, after which 6 out of the 16 contacts were chosen for connection to an IPG for future use during exercise and standing. This meant that knee extension moment could not be tracked with stimulation through all 16 contacts after 6 months. This is largely mitigated because the 6 contacts were specifically selected based on their ability to strongly activate and isolate distinct populations of knee extensors. A similar limitation is that stimulation charge thresholds were tracked through only these 6 contacts after the percutaneous phase ended, and charge thresholds at 9 months and 1 year were derived from moment data rather than EMG measurements. However, it is unlikely that this change in methodology would impact the outcome as the link between CMAP areas and moment generation are well established [94].

C-FINE-Elicited CMAP amplitudes are larger than surface-elicited CMAP amplitudes. However, the opposite relation holds for CMAP areas. This is likely due to limitations in the stimulator used in this study which requires 1 ms between deliveries of stimulation on different contacts. Over 8 contacts, a 7 ms delay develops from stimulation on the first through eighth contacts. This can cause excitation of some populations of motor units while others are in their refractory period, resulting in reduced CMAP areas. When charge required for supramaximal stimulation increases these effects are exaggerated because stimulation of all motor units relies more heavily on charge delivered by multiple contacts which are temporally out-of-phase.

It is not surprising that EMG measured electrodiagnostic changes in the setting of surgery with nerve manipulation and NCE implantation. However, it is remarkable that the EMG findings of fibrillation potentials and SNAP alterations are relatively minor and transient, with minimal clinical relevance. It is also crucial to balance these findings with the marked clinical benefits and restored function obtained with these neuroprostheses. For instance, this standing neuroprosthesis helped this subject achieve long standing times shortly after full system implantation. This research lays the groundwork for determining which electrodiagnostic findings may be expected, and the time-course over which they may resolve. Observing active denervation on EMG more profound or persistent may suggest that NCEs are causing ongoing irritation or nerve compromise. Based on the severity of the changes, the clinical course and medical management of implant recipients can be adjusted, and NCEs removed or replaced accordingly.

## Conclusions

This is the first human trial reporting the year-long clinical electrophysiological changes and rehabilitative improvements in a subject following the application of and stimulation through NCEs. The robust and increasing femoral motor responses, in conjunction with the temporary EMG signs of neurapraxia and irritation perioperatively, establish that the C-FINEs were safely implanted on the proximal femoral nerve trunks with signs of only minor perioperative irritation. Hip flexion and the proximity of the C-FINEs to the inguinal ligaments did not result in entrapment mononeuropathy. These outcomes accompany deployment of a neuroprosthesis enabling standing and exercise 6 years after SCI. For this particular NCE design, these results suggest that C-FINEs may be implanted near joints and other technically demanding areas with minimal and temporary effects on nerve health, although further study would be required for other specific anatomies. There are numerous broader implications for novel NCE design and deployment. Prior to this study it was unclear if subtle changes in electrodiagnostics would require surgical modification or removal of an NCE. These results present the electrodiagnostic findings and time course for their resolution which may be expected in a fully functioning neuroprosthesis. This suggests some of the electrodiagnostic changes which could be considered essentially subclinical compared to those requiring intervention. Moreover, this testing protocol may be employed in the initial deployment of other novel NCE designs to reassure that they are safe and these protocols are potentially applicable to other peripheral nerves anatomies.

## Data Availability

The study protocol, informed consent form, and individual participant data that underlie results reported in this article, after de-identification, are available from the corresponding author on reasonable request for 3 years following publication.

## Abbreviations

C-FINE: Composite flat interface nerve electrode
CMAP: Compound muscle action potential
CWRU: Case Western Reserve University
EMG: Electromyography
IPG: Implanted pulse generator
IST-16: 16-channel implanted stimulator-telemeter
MNCV: Motor nerve conduction velocity
NCE: Nerve cuff electrode
NCS: Nerve conduction studies
RF: Rectus femoris
Sart: Sartorius
SCI: Spinal cord injury
SNAP: Sensory nerve action potential
SEM: Standard error of the mean
VI: Vastus intermedius
VL: Vastus lateralis
VM: Vastus medialis
